# Editorial: The roles of peripheral immune cells and their circulatory effector molecules in neuropsychiatric disorders

**DOI:** 10.3389/fncel.2024.1471683

**Published:** 2024-09-02

**Authors:** Bernhard T. Baune, Eve-Marie Tremblay, Karl Bechter, Li Tian

**Affiliations:** ^1^Department of Psychiatry, University of Münster, Münster, Germany; ^2^Division of Medical Sciences, University of Victoria, Victoria, BC, Canada; ^3^Department of Psychiatry and Psychotherapy II, Bezirkskrankenhaus Günzburg, University of Ulm, Ulm, Germany; ^4^Department of Psychology and Logopedics, University of Helsinki, Helsinki, Finland

**Keywords:** mental health, cytokine, microglia, immunity, neuroinflamamation

It is well acknowledged that alterations in the innate and adaptive immune systems play a crucial role in the development and maintenance of mental illness (Zhang et al., 2023). For instances, aberrant cytokine levels occur, both in the acute phase and in the chronic course, in major psychiatric disorders such as schizophrenia (SZ), bipolar disorder (BD), and major depression disorder (MDD). In particular, interleukin (IL)-6, tumor necrosis factor (TNF) alpha, and high-sensitive C-reactive protein (hsCRP) levels appear elevated in the acute phase and IL-6 during the chronic course across diagnoses (Orbe and Benros, 2023).

Among the myeloid-lymphoid systems, microglia, which are the resident innate immune cells of the central nervous system (CNS), have emerged over the past decade as playing a key role in various mental illnesses, in concert with changes taking place in the periphery (Chaves-Filho et al., 2024). Monocytosis, neutrophilia, and lymphocytopenia are also characteristic for depressive disorders (Chan et al., 2023). In patients with BD, imbalanced ratios and an altered function of T helper (Th) 1, Th2, Th17 cells, and regulatory T cells are particularly evident (Chan et al., 2023), while patients with SZ also show increased lymphocyte and monocyte levels (Orbe and Benros, 2023).

Although the past decades have witnessed a rapid progress in immunopsychiatry, consolidated applications of the relevant research findings in clinical diagnosis and therapeutical interventions of mental illnesses are still limited. Understandably, immune alterations across the increasingly long lifespan of humans are very dynamic and heterogeneous (Chen et al., 2024), and immunity-mediated brain impairments start already from fetal period, due to for example prenatal exposure to adversities such as infections and psychosocial stressors (Chan et al., 2023). Such developmental imprint on the immune system can further extend postnatally to impact not only early childhood and puberty but also late adulthood and old age (Mposhi and Turner, 2023). Hence, dissecting specific biological factors shaping immune functions at different disease stages of mental illnesses is still crucially needed for developing better diagnoses and interventions of these disorders. In this Research Topic of articles, the importance and relevance of peripheral and CNS immune cells and their regulatory effector molecules are highlighted by five different articles.

Firstly, Stanley et al. provided an insightful review about the role of microglia in “neuroinflammation” (i.e., inflammation taking place in the CNS) by focussing on three colony stimulating factors (CSFs): CSF-1/IL34, G-CSF, and GM-CSF. Interestingly, these findings are based on discoveries about the role of the CSF-1-receptor-related to the neurodegenerative disease leukodystrophy (Hume et al., 2020). The overall high complexity including direct actions as well as regional differences of CSFs signaling in the brain was outlined in detail and well-illustrated in high-quality figures. The authors added with their review to the debate on whether neuroinflammation and/or neurodegeneration contribute to the development of mental illness by addressing important key points from a molecular-cellular perspective. The clinical challenge to diagnose and define mild neuroinflammation possibly involved in severe mental disorders gets additional insights by this work, which appears to be along the line of the parainflammation (i.e., an intermediate immune response between basal and inflammatory states) concept proposed by Medzhitov (2008). This line of research may help identify *in vivo* the differences between milder forms of neuroinflammation and neurodegeneration, which is of clinical importance (Bechter, 2020). The present advances of these important signaling factors provide new therapeutic options, as argued by the authors.

Secondly, Schütze et al. described with broad clinical experience and diagnostic procedures in septic encephalopathy (SE), which as the author argued could be better defined as sepsis-associated encephalopathy since bacteria are not found in the normal brain. Surprisingly, SE diagnosis is rather difficult to establish in a clinical setting and differential diagnoses of meningitis and encephalitis are obvious, which require cerebrospinal fluid examination. In contrast, neuroimaging by computed tomography is rather insensitive, and magnetic resonance imaging is of preferred use in this instance as it is more sensitive to identify brain oedema and white matter lesions, both of which can be indicative of neurodegeneration. In addition, elevated specific circulatory humoral factors may support these imaging findings. The authors provided valuable insights into such diagnostic parameters of SE in light of neuroinflammatory and neurodegenerative processes in both humans and animal models.

Thirdly, Zhang et al. presented a case control study on 50 patients diagnosed with SZ as compared to healthy controls, investigating blood-based concentrations of neurotransmitters, macrophage/glia markers, cytokines, and content of poly-unsaturated fatty acids (PUFAs) and membrane fluidity in erythrocytes. Their results indicated a lack of clear abnormality of T cells in SZ, but a tendency of proinflammatory signature in association with decreased n-3-PUFAs and dysfunctional neurotransmitters as well as decreased membrane fluidity. This work adds to several previous research findings that point toward a slight proinflammatory blood status in SZ, which could be interpreted as state or/and trait markers. Moreover, the authors suggested that the underlying systems may also be relevant in the context of environmental factors like diet as well as through genetic liability. They also suggested that reduced membrane fluidity of erythrocytes (and potentially other cells) may be relevant as a potential trait marker on one hand, but also a state marker on the other hand, since neuroleptic treatments possibly influenced membrane fluidity. Indeed, both human and animal studies have demonstrated anti-inflammatory effects of neuroleptic medication in the periphery as well as the brain (MacDowell et al., 2013).

Fourthly, Kim et al. reported a study in mice during pregnancy with the aim to investigate alterations in the brain and periphery of dams after subcutaneous dexamethasone injections during gestational days 16–18. The new insight presented is that dexamethasone induced anxious-depressive behavior 3 weeks after birth in newborn pups without any changes of the brain-immune-inflammatory system, except the enhanced microglial reactivity in the hippocampus and, more surprisingly, decreased IL-10 in the peripheral lymph nodes but normal IL-10 levels in the blood and brain. Interestingly, these abnormalities at postnatal 3-week appeared to be normalized at week-10. These results are important findings that contribute to the sparse literature on the interaction between peripheral and central immune cells in an early immune-inflammatory depression-anxiety model.

Finally, Khantakova et al. provided a review of the literature following PRISMA recommendations in rodent models of neonatal depressive-like behaviors, examining associated changes in brain and immune functions, pertaining to microglia, astrocytes, neurogenesis, oxidative stress, the hypothalamic pituitary adrenal (HPA)-axis, and cytokines. The authors defined a neonatal immune activation paradigm, in which immune challenge takes place during early postnatal development, which is comparable to findings in human depressive disorders. The authors concluded that alterations in brain cells, especially reactive microglia, and imbalance of cytokines in the brain but not in the blood, as well as a hyperreactive HPA-axis, accompanied by an increased vulnerability to developing depressive behavior later in life, showed a broad consistency in the literature.
